# Reflection on feasibility and usability of interactive online international exchange program for occupational therapy students

**DOI:** 10.1007/s44217-023-00031-4

**Published:** 2023-01-31

**Authors:** Natsuka Suyama, Kaoru Inoue, Supatida Sorasak, Chirathip Thawisuk, Masaru Watanabe

**Affiliations:** 1 Department of Occupational Therapy, Graduate School of Human Health Sciences, Tokyo Metropolitan University, 7-2-10 Higashiogu, Arakawa-Ku, Tokyo, 116-8551 Japan; 2 Occupational Therapy Division, Faculty of Physical Therapy, Mahidol University, Salaya, Thailand

## Abstract

The COVID-19 pandemic affected international exchange programs due to travel restrictions. There are few reports of online international exchange programs in medical area and no report of interactive activities among students together for several months. To the best of our knowledge, we needed to seek the feasible approach to conduct online exchange program. We experienced online collaboration works to promote internationalization in occupational therapy (OT) undergraduate education. The aim of this study is to examine the feasibility and usability of an interactive online international exchange program, and discuss its challenges and advantages. The three-month program was newly developed, and aimed at cultivating an international perspective, understanding OT, and learning basic research skills. Students’ interests and the development of their motivation were considered in the program consisting of synchronous and asynchronous lectures, a collaboration research project, group work, and report assignments. Through the program, students were satisfied with the program contents and had the precious experience to develop international relationships. The online exchange program provided students with the opportunity to experience and learn OT education internationally at an economical cost, and to achieve program goal during the regular semester. In this program, students not only acquired knowledge in their professional field, but also cultivated their international communication skills in English from small group activities and social activities. Considering various communication methods was an important aspect in the virtual environment leading to a successful program. In medical education, an online program can be an effective and practical measure for international exchange.

**Supplementary Information:**

The online version contains supplementary material available at 10.1007/s44217-023-00031-4.

## Introduction

Educational institutions have faced challenges since the COVID-19 pandemic because many curriculums were expected to provide in-person instruction. Amidst the pandemic, many universities were required to provide distance learning education. In the medical educational field including occupational therapy (OT), although education in which practical training in-person is an important part of the curriculum, both lectures and practical training had to be provided online. During the pandemic, studies on virtual class learning have been conducted and reported [1–3]. Furthermore, international exchange programs also faced challenges due to travel restrictions. The objectives of international exchange programs include not only acquiring academic knowledge, but also experiencing a different culture. Hence, to promote international exchange in the medical educational field, international academic and cultural programs needed to be considered for online style even during the pandemic. Our university usually has short-term exchange programs abroad as an elective subject opening to students who want to take. However, the pandemic restricted travel, and the existing program was cancelled without transitioning to an online program. Instead of in-person program, we, Department of Occupational Therapy, Tokyo Metropolitan University(TMU), Japan, and Occupational Therapy Division, Mahidol University(MU), Thailand, had the opportunity to create a new three-month online exchange program in 2021. In the medical educational field, there are a few reports of online international programs such as virtually visiting a medical institution in another country [4, 5]. However, there are few reports of international exchange programs in which students participate from two or more universities and conduct activities together for several weeks [6, 7]. To the best of our knowledge, there is no report promoting a program with interactive communication for a period of several months.

Blended learning that combines face-to-face and online contents, and online distance education are not novel methods in higher education, even before the pandemic [8–10]. Such programs have been intentionally created as online learning programs. However, during the pandemic, students and lecturers were suddenly faced with changing learning contents originally expected as in-person exchange programs. Therefore, an interactive international exchange program could pose challenges but also provide further opportunities to promote internationalization using information technology even after the pandemic. To produce effective instruction, the program should have good goals, contents, and reasonable resources. All instructional design models require analysis, design, development, implementation, and evaluation phases to develop education [11]. In addition, the Attention, Relevance, Confidence, and Satisfaction (ARCS) model of motivation developed by John Keller [12, 13] proposes significant concept on students’ attention to class, the relevance to participants, students’ confidence in the class, and their satisfaction. ARCS model has been widely accepted and used to promote students’ motivation and participation actively in the class [14, 15], and the online program was conducted effectively based on ARCS model [16]. When a program is created based on the ARCS model considering participants’ attitudes, participants would be supported to join the program actively and to experience international communication even through virtual contents.

Even though the ARCS model was supported as an instructional model for online program [16], there is no report of an international exchange program with interactive communication for a period of several months in medical education field. The aim of this study is to examine the feasibility and usability of an interactive online international exchange program and discuss the challenges and advantages of virtual exchange program posed by travel restrictions due to COVID-19. This study contributes to the development of virtual learning education in the medical field including OT education.

## Materials and methods

### Creating online instructional strategies

A new program not based on the existing program contents was needed considering the pandemic situation. This program was separate from the regular curriculum and not given a credit. The goals of the program were to cultivate an international perspective, to understand OT from lectures and group discussions, and to learn basic research and presentation skills. The program was created based on the ARCS model [12]; lecturers selected appropriate topics to achieve the program goals. In addition to lecture contents, assignments and activities were organized to improve students’ understanding. All contents were created considering students’ academic progress and interests to maintain their motivation. This program was mainly created by TMU (Japan) lecturers (NS and KI) and MU (Thailand) lecturer (SS) under agreement and students from both universities joined the same program.

The online program was advertised to targeted students (TMU, 44 students; MU 33 students). Students decided to join of their own accord after an English group interview by lecturers to determine students’ language proficiency. A total of 17 OT students participated in the program (seven from TMU and 10 from MU), which ran from June 10 to September 15, 2021. During the program schedule, TMU students were in the first semester of their third year, and MU students were in the second semester of their second year, transitioning to the first semester of their third year. The students had almost the same academic learning progress level except for clinical placement.

### Program contents and tools

TMU and MU arranged a three-month online exchange program. It consisted of synchronous and asynchronous lectures, a collaboration research project, group work, and report assignments. The first session was an orientation and ice-breaker to get to know each other, and the final session was students’ presentations of their collaboration research projects. The communication language was English. Table 1 shows the content details.

**Table 1:** Program contents from June 10 to September 15, 2021

Session	Date	Time	Contents	
1	June 10, 2021	10:30 am–12:00 pm (UTC + 0900) 8:30 am–10:00 am (UTC + 0700)	Orientation, introduction	
2	June 17, 2021		Medical and welfare system	Medical and welfare system in Japan, Long-term care insurance (by TMU lecturer)
3	June 24, 2021			Health and welfare system in Thailand (by MU lecturer)
4	July 1, 2021		OT education	OT MU education and practice placement (by MU lecturer)
5	July 8, 2021			OT TMU education and practice placement, OT working area in Japan (by TMU lecturer)
6	July 15, 2021		Research and university project	Research skills and research at TMU (by TMU lecturer)
7	June 22, 2021			MU OT current project (by MU lecturer)
8	July 23 to August 12, 2021	On-demand	Occupational therapy in Asian countries (by TMU lecturer and TMU international students)	
9	August 5 to 17, 2021	On-demand	Occupational therapy perspective on Covid-19 pandemic (by MU lecturer)	
10	August 18, 2021	4:00 pm–5:30 pm (UTC + 0900) 2:00 pm–3:30 pm (UTC + 0700)	OT practice in clinical setting	OT practice in Japan and introducing assistive products in a clinical setting (by TMU lecturer)
11	August 25, 2021			Occupation-based practice in mental health (by MU lecturer)

Three weeks (collaboration research project)			
12	September 15, 2021	4:00 pm–5:30 pm (UTC + 0900) 2:00 pm–3:30 pm (UTC + 0700)	Students' project presentations

For the assignment in each lecture session, short discussions or report assignments were required from the students. After finishing half of the sessions, students picked several topics for their collaboration research project, and then the lecturers grouped students in groups of four to five and assigned research topics based on their interests before the three-week session break. The project member was mixed TMU and MU students in each group. During this period, students were encouraged to work together through online tools based on what they learned professional knowledge and research skills during program.

The program was arranged by coordinator lecturers from each university. A video application system (Zoom: Zoom Video Communications, Inc., USA) was used for interactive live lectures, and a free cloud service (Google site: Google LLC, USA) was used to share information and materials and establish an original website. Lecturers and students were able to find program contents, and provide/get handout and recorded lectures materials for preparing and reviewing. As supplemental tools, a social network service (LINE: LINE Corporation, Japan) and cloud platform service (Slack: Slack Technologies, Inc., USA) were introduced to enable communication outside of class. During program period, students were able to work together for their assignment through those tools. Students were encouraged to join all online lectures and communicate with each other outside of lectures. As an optional social activity, lecturers paired students from each university, and the students were invited to create a short film to introduce each culture.

### Program evaluation methods from students

In the study, program evaluation from students of interactive online exchange program was examined from seven TMU students’ perspective and their activity products. This study was performed in line with the principles of the Declaration of Helsinki. Approval was granted by the Ethics Committee of Tokyo Metropolitan University (Approval Number 21093). Informed consent was obtained from all individual students included in the study. Students were required to complete questionnaires on the program contents and International Posture (IP) within two days after the program (see supplemental material). IP was originally developed for the study of the learning motivation of a foreign language learner, and evaluates the attitude to international society [17, 18]. Yashima [19] published a Japanese version of IP, which consists of 28 items and 5 factors, on a 6-point likert scale [scare 1 (negative) to score 6 (positive)] [20]. The five factors are *intercultural approach-avoidance tendency*, *interests in international vocation*, *ethnocentrism (reaction to different customs/values/behaviors)*, *interest in foreign affairs*, and *having things to communicate about (willingness to communicate to the world)*. The IP score reveals the concept of the attitude between the learner and international connection using a foreign language. These attitudes could reflect a student’s perspective of international exchange and working internationally in the OT field in the future. In addition to questionnaires on the program contents and IP, students were asked on program reflection as “How was this program?” in free comments just before the last session.

## Results

### Student activities and communication outside of class

All students joined the online lectures and group discussions, and in the final collaboration work, TMU and MU combined student groups presented four research projects. They prepared their work outside of class. The following research projects were conducted: *Mental health and life balance*, *Aging society*, *Home and environmental adaptation*, and *Assistive devices in pediatrics*. In addition, they had a cultural exchange program for mutual understanding outside of class. Student groups created short films as optional activities. They introduced and made films on traditional culture and food, young culture, lifestyle, and school culture.

Through the program, students learned about OT in each country and basic research skills from the lectures, assignments, and collaboration projects. Students were always free to access any lecturers to ask questions or for advice. The lecturers did not need to provide much support to the students because they managed their work well by themselves.

### Program evaluations from students

Answering the questionnaires from TMU students was voluntary; five students of seven answered the questionnaires, and all seven students provided reflections on the program. Table 2 shows the students’ evaluations of the program contents. The students answered that the program was useful and of good quality. For the IP score, we analyzed the average of factor among participants. *Intercultural approach-avoidance tendency* was 5.22, *interests in international vocation* was 4.63, *ethnocentrism (reaction to different customs/values/behaviors)* was 3.16, *interest in foreign affairs* was 3.50, and *having things to communicate about (willingness to communicate to the world)* was 3.40. From the answer result, high- and low-scoring items was picked up. In the factor *intercultural approach-avoidance tendency*, students tended to reply *I want to make friends with international students* and *I want to participate in a volunteer activity to help foreigners living in the surrounding community*. In the factor *interests in international vocation*, they responded that *I’m interested in overseas news* and *I’m interested in an international career*. Students tended to note that *it is enjoyable to do work together with a person having different custom and values* as a *reaction to different customs/values/behaviors*. In the factor *having things to communicate about*, they responded that *I have a lot of things to talk about with foreign friends*. Students did not show a strong tendency for *I have ideas about international issues, such as environmental issues* and *I have my own opinion about international issues*.

**Table 2:** Student evaluations of the program contents

How was the overview of the program?	N = 5(%)	Were the program contents useful?	N = 5(%)
Very good	3(60)	Very useful	4(80)
Good	2(40)	Useful	1(20)
Not very much	0(0)	Not very much	0(0)
Not at all	0(0)	Not at all	0(0)

How was the level of the contents?	N = 5(%)	Do you want to join the program again next time?	N = 5(%)
Too easy	0(0)	Yes	4(80)
Just right	2(40)	No	0(0)
Too difficult	3(60)	Maybe	1(20)

Participants’ reflections were generally positive. The following are some examples: *“International exchange program was a valuable experience, and very valuable opportunity”*; *“To know OT in various countries was good”*; *“It was a precious experience to meet Thai students studying toward the same goal. Working together in this program was a wonderful time to learn from various perspectives. MU students’ positive attitude allowed me to express my opinion in English”*; and *“It’s a great opportunity to learn about OT not only in other Asian countries but in Japan.”* In addition to the contents, some students expressed that communicating solely in English was challenging but that the program encouraged them to improve their English: *“I answered that the contents were too difficult, it means that the English was hard, but the contents was at a good level, so I was encouraged to learn English more”*; *“This exchange program with Thai students was a precious and enjoyable time. I’d like to keep learning English”*; and *“I want to learn more English communication.”* Furthermore, social communication and activities promoted friendships: *“We were assigned to buddies off-campus, and some group works were helpful to get to know each other and make friends more easily. That was good”*; *“It was a great experience to interact with people of the same age in other countries. I am glad I participated”*; and *“I’m so happy to be in this program, to work with others, and to learn things I didn’t know before.”* There was no negative feedback about the program contents, although some students had difficulties adjusting to the schedule outside of class to complete assignments with Thai students.

## Discussion

COVID-19 affected international exchange programs due to travel restrictions; however, we experienced international collaboration work online. Even though an online program differs from an onsite experience, students had a valuable experience and opportunity to understand OT in another country and different culture. We discuss the feasibility and usability of the international program from three perspectives: program contents, students’ attitudes, and communication methods. In addition to these points, the implication of an online international exchange program is discussed.

First, the program contents were created by the program project team, considering students’ academic progress levels and their requests. Therefore, students were able to maintain their motivation to join for three months and they all joined the program. The ARCS model suggested a measure for maintaining motivation when learners face challenges [12]. The model notes that educators should lead learners to options to handle challenges in a new environment such as a virtual setting. In our program, several communication tools and well-organized contents schedule considering students’ interest were provided, and participated students were able to work together, given opportunity of group work and joint project. Students managed their activities by themselves based on the core contents of the lecture schedule, and developed their learning with high motivation by themselves.

Second, from students’ evaluations and attitude toward the program, the program was generally appropriate and provided students with a valuable experience, even though some students struggled with the English communication. English proficiency itself did not affect motivation to join the program; rather, students wanted to improve their English to communicate more effectively with their friends from the other country. Learning topics, different cultural experiences than usual, and social activities among peers from the same generation might improve students’ motivation. The IP score showed that students enjoyed making friends and joining activities rather than having an interest in being involved in international issues. This might be a case of social enjoyment or personal connection rather than developing international affairs; thus, students may not develop an international attitude. There could be several reasons for this, such as that the program contents mainly focused on OT rather than understanding other cultures. The virtual setting was obviously not as realistic as the onsite experience; therefore, students may not have been able to deepen their experience to their expected international works. In this study, the questionnaire was conducted once after the program was completed; we are not able to see a change in attitude toward international perspective, and the number of data were very small as quantitative data.

Third, one of the challenges in a virtual program is communication methods. Interactive communication or group work in a virtual environment is more difficult than lecture-style contents. The goals of meeting online should be clear and good platforms should be managed accordingly. In this program, when students were assigned group work, the goal setting and schedule were very clear; thus, students were able to organize their work and achieve their goals. The program website was useful to share information and follow the program progress. In addition, several communication tools (LINE, Slack) were used to exchange ideas. Time management and technological literacy are significant factors for students in online programs [21]. Thus, an organized program and familiar tools to promote communication in this program was very important and useful. Furthermore, social media could assist both communication and active learning in medical education [22, 23]. The effectiveness of social media or communication tool package may help to improve students’ work more.

Through the program, students understood OT in each country and developed an interest in the other culture. These experiences encouraged students to engage in more English international communication. Moreover, students cultivated basic research skills from lectures and the research project. Therefore, this program achieved its purpose and students maintained their motivation to join the program. Students actively participated in the program for various reasons. One reason could be the program contents which helped them maintain their motivation [24]. The program was established based on clear goals, had a well-organized schedule and communication opportunities among participants. Students in this program could also have been highly motivated because they were willing to apply. Therefore, lectures only needed to provide core learning content, and participants were able to manage their work by themselves [25]. Furthermore, this program was arranged with not only lecture contents but also activities and tools promoting communication such as social activities and small group work; these supports led to a successful program even though it was conducted in an online environment.

### Challenges and advantages of the online program

When the feasible online program is planned, some challenges and advantages should be discussed. In an online program, one of the biggest issues is the network environment [7, 26]. Although the internet environment in our case was mostly stable and had a good connection, maintaining stability may be a significant problem depending on the area and socio-economic factors [1]. In addition, technological literacy and competency could be challenges for both students and lecturers [21]. Technological literacy affects communication among participants and influences program satisfaction. Therefore, educator could cooperate with technological support service in educational institution to improve the sufficient program quality. Moreover, in the exchange program, synchronous classes could be challenging due to time differences and thus, program session and assignments should be carefully arranged [6]. In our program, the time difference was two hours, so it was not difficult to adjust; however, the program was conducted for three months long. We needed to consider the semester schedule and regular works in each university.

The online exchange program had several advantages. First, the cost was low because there was no need to travel. In addition to the cost, even during the semester, students can simultaneously have the experience of international exchange and develop their knowledge based on both domestic and international knowledge. An exchange program with travel involves high travelling and accommodation costs, and students have to abandon their domestic curriculum for some period. Therefore, an online program can save money and time.

### Implications and further improvement

In OT education, an online program can be an effective and feasible measure for international exchange. The medical education curriculum is busy because of being required to take many subjects and practical placement. Even during the regular semester schedule, an online program enables students to join the exchange program and develop their international perspective. In addition, there are many innovation tools to communicate from a distance, for example, devices and web services such as applications and cloud platform services. These tools are very helpful to improve the further quality of online programs in the future. Students from the new generation are also familiar with these new technologies and some faculty students enjoy creating virtual reality and tour contents and learning from them [27]. Thus, perhaps introducing digital tools could promote students’ active learning attitudes both online and in in-person classes. The possibility of online program by using new technology has to be developed when students and teaching staff learn and get to use to the online application and devices for smooth communication and conducting activities. Still, the communication environment is limited compared to in-person communication; thus, small group sessions and well-organized contents are essential. As a limitation of this study, the influence of the program contents on students’ attitudes toward international interests is still unclear due to the small number of quantitative data. Furthermore, students had highly motivation to join; thus, the program progressed well. However, when a general program with credit is created, the communication method and individual competency should be considered. The further research of large data and depth data collection such as interview method will be needed to examine educational program, in addition to data size, IP questionnaire to evaluate students’ attitude toward international perspective has not been updated for years and not specific scale for online program, therefore, scales to evaluate the change of international attitude by the online international exchange program should be considered in the future research.

## Conclusion

The COVID-19 pandemic affected international exchange programs due to travel restrictions. However, we experienced international collaboration in OT education via an online program. The program was created considering students’ interests and the development of their motivation, and consisted of synchronous and asynchronous lectures, a collaboration research project, group work, and report assignments. Students were satisfied with the program contents and had the precious experience of international exchange. In this program, students were able to cultivate an international perspective, to understand OT from lectures and group discussions, and learn basic research and presentation skills. Even though there were some challenges such as communication, the exchange program via information technology offered students the effective learning opportunity to experience OT education internationally. In addition, the feasible program management and schedule at a low cost was beneficial aspect of online program. In medical education, an online program can be an effective and feasible measure for international exchange.

## Supplementary Information

Below is the link to the electronic supplementary material.

Supplementary file1 (DOCX 16 KB)

## Data Availability

The data used to support the findings of this study are included within the article. However, for more information, requests for access to these data should be made to Ms. Chihiro Sasaki (kcwgt0111@yahoo.co.jp).
