# Supplementary material for: Reflection on feasibility and usability of interactive online international exchange program for occupational therapy students
Source: Discov Educ. 2023 Jan 31;2(1):7. doi: 10.1007/s44217-023-00031-4 (PMC9887562; doi:10.1007/s44217-023-00031-4)
Supplement: Supplementary file 1 — Supplementary file1 (DOCX 16 KB) [file 44217_2023_31_MOESM1_ESM.docx]

**Supplementary Material**

Questionnaire of program feedback

1. How was the overview of the program?

Very good/Good/Not very much/Not at all

1. Were the program contents useful?

Very useful/Useful/Not very much/Not at all

1. How was the level of the contents?

Too easy/Just right/Too difficult

1. Do you want to join the program again next time?

Yes/No/Maybe

1. If you have any comments on program contents and structure, please write here.

International Posture (IP) questions (*negatively worded items, factors structure is not revealed to respondents)

Answer in scale from one (I strongly disagree) to six (I strongly agree)

*Intergroup Approach-Avoidance tendency*

1. I want to make friends with international students studying in Japan.
2. I try to avoid talking with persons from abroad if I can. *
3. I would like to talk to an international student if there was one at school.
4. I wouldn't mind sharing an apartment or room with an international student.
5. I want to participate in a volunteer activity to help people from abroad living in the surrounding community.
6. I would feel somewhat uncomfortable if a person from abroad moved in next door. *
7. I would help a person from abroad who is in trouble communicating in a restaurant or at a station.

*Interest in International Vocation or Activities*

1. I would rather stay in my hometown. *
2. I want to work in a foreign country.
3. I want to work in an international organisation such as the United Nations.
4. I'm interested in an international career.
5. I don't think what's happening overseas has much to do with my daily life. *
6. I’d rather avoid the kind of work that sends me overseas frequently. *

*Ethnocentrism (Reaction to different customs/ values/behaviours)*

1. I sometimes feel discomfort in attitude of a person from abroad.
2. I prefer associating with persons having similar value to person with having different value.
3. I think that working together with people having different culture and values is enjoyable. *
4. I want to work with persons with similar thought and value.
5. I’m not good at persons having different habit and value from mine.

*Interest in International Affaires*

1. I often read and watch news about foreign countries.
2. I often talk about situations and events in foreign countries with my family and/or friends.
3. I have a strong interest in international affairs.
4. I'm not much interested in overseas news. *

*Having Things to Communicate to the World*

1. I have thoughts that I want to share with people from other parts of the world.
2. I have issues to address with people in the world.
3. I have opinions about international issues and events, such as environmental issues and north-south issues.
4. I have no idea what I should talk to persons from abroad. *
5. I have no clear opinions about international issues. *
6. I have a lot of things to talk with friend in foreign countries.
